# Understanding Direct Powder Extrusion for Fabrication of 3D Printed Personalised Medicines: A Case Study for Nifedipine Minitablets

**DOI:** 10.3390/pharmaceutics13101583

**Published:** 2021-09-29

**Authors:** Sergio A. Sánchez-Guirales, Noelia Jurado, Aytug Kara, Aikaterini Lalatsa, Dolores R. Serrano

**Affiliations:** 1 Department of Pharmaceutics and Food Science, Facultad de Farmacia, Universidad Complutense de Madrid, 28040 Madrid, Spain; sanchez.sergio@uces.edu.co (S.A.S.-G.); njurado@ucm.es (N.J.); akara@ucm.es (A.K.); 2 Biomaterials, Bio-Engineering and Nanomedicine (BioN) Lab, Institute of Biomedical and Biomolecular Sciences, School of Pharmacy and Biomedical Sciences, University of Portsmouth, Portsmouth PO1 2DT, UK; katerina.lalatsa@port.ac.uk; 3 Instituto de Farmacia Industrial y Galénica, School of Pharmacy, Universidad Complutense de Madrid, 28040 Madrid, Spain

**Keywords:** 3D printing, fused-deposition modelling (FDM), hot-melt extrusion, HME, direct powder extrusion, minitablets, nifedipine, cardiovascular diseases

## Abstract

Fuse deposition modelling (FDM) has emerged as a novel technology for manufacturing 3D printed medicines. However, it is a two-step process requiring the fabrication of filaments using a hot melt extruder with suitable properties prior to printing taking place, which can be a rate-limiting step in its application into clinical practice. Direct powder extrusion can overcome the difficulties encountered with fabrication of pharmaceutical-quality filaments for FDM, allowing the manufacturing, in a single step, of 3D printed solid dosage forms. In this study, we demonstrate the manufacturing of small-weight (<100 mg) solid dosage forms with high drug loading (25%) that can be easily undertaken by healthcare professionals to treat hypertension. 3D printed nifedipine minitablets containing 20 mg were manufactured by direct powder extrusion combining 15% polyethylene glycol 4000 Da, 40% hydroxypropyl cellulose, 19% hydroxy propyl methyl cellulose acetate succinate, and 1% magnesium stearate. The fabricated 3D printed minitablets of small overall weight did not disintegrate during dissolution and allowed for controlled drug release over 24 h, based on erosion. This release profile of the printed minitablets is more suitable for hypertensive patients than immediate-release tablets that can lead to a marked burst effect, triggering hypotension. The small size of the minitablet allows it to fit inside of a 0-size capsule and be combined with other minitablets, of other API, for the treatment of complex diseases requiring polypharmacy within a single dosage form.

## 1. Introduction

Since the FDA approval of Spritam^®^, there has been a growing interest in the application of 3D printing in pharmaceutical sciences [1]. 3D printing is an innovative technology that allows the fabrication of personalised medicines to enable a tailored pharmacotherapy instead of a “one size fits all” approach [2,3,4]. 3D printing allows for tailoring of dosage forms to patients’ and disease needs such as age, dose, release profile, colour, texture, taste, and size [5]. Their ability to allow for dose individualisation enables 3D printed dosage forms to ensure efficacy combined with minimal side effects [6]. 3D printing makes feasible the incorporation of multiple active pharmaceutical ingredients (API) into one dosage form that can result in enhanced adherence and better and safer pharmacotherapy in polymedicated patients [7].

Based on WHO statistics, cardiovascular diseases (CVDs) are the leading cause of death globally, taking an estimated 17.9 million lives each year, which represents 31% of all deaths worldwide. CVDs are a group of disorders of the heart and blood vessels and include coronary heart disease, cerebrovascular disease, rheumatic heart disease, and other conditions. Four out of five CVD deaths are due to heart attacks and strokes, and one-third of these deaths occur prematurely, in people under 70 years of age [8]. Although medications for CVDs are effective and can yield very substantial health and economic benefits, these advantages are often not realised, as approximately 50% of patients do not take their medications as prescribed [9]. The annual cost of non-compliance due to medication-related hospital visits may be as high as $100 billion [10]. Lack of adherence can be overcome by combining complex treatments within the same solid dosage form.

There are several types of 3D printing technologies which, according to the 3D printing classification of the American Society for Testing and Materials (ASTM) [11], can be classified into seven categories: vat photopolymerisation, material jetting, binder jetting, material extrusion, powder bed fusion, sheet lamination, and directed energy deposition. Among these 3D printing technologies, material extrusion (in particular, fused deposition modelling (FDM)) is the most commonly used in pharmaceutical sciences due to the wide availability and low cost of printers [12]. However, before printing by FDM is materialised, the manufacture of a drug-loaded filament by holt-melt extrusion (HME) is necessary [13]. HME can increase the likelihood of drug degradation due to high temperatures applied during the process. Also, there is a limitation on the number of excipients and drugs capable of resulting in filaments with mechanical and physical properties suitable for FDM. Furthermore, the batch sizes for extrusion are relatively large, while the optimisation of the formulation is complex, which hinders its translation for precious APIs in clinical practice [12].

Direct powder extrusion involves the feeding of a powder mixture into the printer, heating, and extrusion of the API-excipient mixture, and printing by deposition according to the designed geometry. The need for the manufacturing of drug-loaded filaments by HME with the appropriate mechanical and physical properties can be omitted by employing 3D printers that allow direct powder extrusion of the API mixture. Additionally, direct powder extrusion overcomes limitations around drug loading that usually occur with conventional HME, as the latter requires a larger amount of excipients to avoid fabricating too-brittle or too-flexible filaments that cannot be loaded into an FDM printer. Therefore, this strategy can be of immense value in clinical settings with less manufacturing expertise, and it can simplify the 3D printing process, transforming the current two-step process (HME followed by FDM) into an innovative and more cost-and-time-effective single-step 3D printing manufacturing process [12]. However, direct powder extrusion of pharmaceuticals has not been widely explored to provide necessary understanding as a basis for uptake of the technology in manufacturing of 3D printed dosage forms.

The combination of API with suitable excipients and direct extrusion can also enhance the dissolution profile of poorly water-soluble BCS Class II drugs such as nifedipine (NFD). NFD is currently commercialised as modified and extended-release tablets, and as an oral solution with a wide range of doses, which indicates the need for dose personalisation [14]. We have previously shown that spherical minitablets of NFD have been successfully manufactured using FDM coupled with HME, and demonstrated NFD controlled release in simulated gastrointestinal fluids from these 3D printed dosage forms [13]. The hypothesis underpinning this work is that direct powder extrusion can more successfully be applied in the development of personalised medicines to treat CVDs in a single-step process than HME followed by FDM. This study demonstrates the design and manufacture of NFD minitablets by a direct powder extrusion technique that can be easily adjusted to the patient’s dose needs by just adjusting the tablet height. A quality by design approach was followed to find the optimal formulation and understand the direct powder extrusion process. Minitablets of NFD were fully characterised and compared with commercially available formulations in terms of their in vitro dissolution profile.

## 2. Materials and Methods

### 2.1. Materials

NFD was purchased from Industria Chimica Italiana (>95%, Bergamo, Italy). Hypromellose acetate succinate (HPMCAS) (AQOAT^®^ LG grade) was a gift from Shin-Etsu (Tokyo, Japan). PEG 4000 and magnesium stearate were purchased from Sigma-Aldrich (Madrid, Spain). Kollidon VA 64 (KVA64) was a gift from BASF (Ludwigshafen, Germany). Klucel hydroxypropyl cellulose (HPC, 95 kDa) LF grade was kindly donated by Ashland (Madrid, Spain). Solvents were HPLC grade and were purchased from Proquinorte (Madrid, Spain). Any other reagents were used without further purification and were of analytical grade.

### 2.2. Methods

#### 2.2.1. Quality by Design: Formulation Optimisation

A quality by design approach was used to find the optimal ratio between excipients and APIs. A 23 simple factorial design was performed using Design-Expert 10 software (Stat-Ease Inc., Minneapolis, USA). The target of this DoE was to optimise the composition of a minitablet containing NFD with controlled drug release. The impact of different excipients employed in the manufacture of 3D printed tablets (PEG 4000, HPC, HPMCAS, KVA64) on the percentage of drug released at 6 h and content uniformity were evaluated.

The amount of API per tablet was kept constant at 25% *w*/*w*. Magnesium stearate (1%) was added to all the tablets to ensure adequate powder flow. The effect of the following three different factors was investigated: (i) the amount of plasticiser (PEG 4000), ranging between 5–15% *w*/*w*, (ii) the amount of hydrophilic excipient (HPC), ranging between 20–40%, and (iii) the type of modified-release polymer, HPMCAS or KVA64. The percentage of the modified-release polymer was varied accordingly, up to 100% of the dosage form weight. Once the polynomial regression models were calculated, the optimisation of the formulation was performed, targeting the greatest content uniformity and the lowest drug release at 6 h, to ensure a 24 h release profile.

#### 2.2.2. Direct Powder Extrusion 3D Printing

##### Preparation of the Formulations

Mixtures of NFD, PEG 4000, HPC, KVA64 or HPMCAS, and magnesium stearate were used as formulations for subsequent 3D printing with direct powder extrusion. Each formulation was prepared at a 20 g batch size and was printed at 165 °C to avoid the melting of NFD (which melts above 170 °C), which can result in degradation, but allows the achievement of a mixture with adequate fluidity to be printed. KVA64 or HPMCAS were used to create a modified release polymeric matrix. PEG 4000 was included as a plasticiser, and magnesium stearate as a lubricant to promote the homogenous transit of the powder mixture for printing. Ball milling (IKA Ultra-Turrax^®^ Tube Drive Disperser) was used to blend the feed powder mixture for 2 min at 6000 rpm after which the mixture was sieved through a 12-US mesh (1.68 mm) screen, before 3D printing. Formulations were protected from light with foil under desiccated conditions, to avoid drug degradation until further analysis was performed.

##### Geometry Design and 3D Printing Settings

The minitablet geometry was designed using a computer-aided design (CAD) software (Tinkercad software v.1, Autodesk 2019, Barcelona, Spain) to create a .stl file compatible with the Repetrel v. 4.101 Software (Norcross, GA, USA). The tablets were designed with a cylindrical shape of 7 mm in width and with a defined height of 2.38 mm. The minitablets were designed to contain 20 mg of the active ingredient (NFD). The total surface area of each minitablet was calculated using Autodesk 3ds Max 2021 (Autodesk, Inc., San Rafael, CA, USA) to be 108.7 mm^2^ [13].

An Engine SR Hyrel FDM 3D printer (Norcross, GA, USA) was used to print the tablets. The powder was filled in a TAM-15^®^ extruder with a 1 mm extrusion nozzle aperture. The platform temperature was set at 70 °C and the printing temperature was set at 165 °C. The print and travel speed were both set at 10 mm/s. The first layer height was set to 0.23 mm and the other layers to 0.12 mm of height. Lastly, the infill percentage of the shape designed was set at 100%.

#### 2.2.3. Content Uniformity and Mass Uniformity

Minitablets (*n* = 3) obtained by direct powder extrusion were weighed and dissolved in a volume of ethanol: water (9:1 *v*/*v*). Absorbance was measured at 240 nm (λ_max_) by ultraviolet spectroscopy (V-730, Jasco, Tokyo, Japan). A calibration curve between 5–50 µg/mL was performed under the same conditions. A good correlation between drug concentration and absorbance was obtained within this range (R^2^ = 0.9999). Minitablets (*n* = 10) were weighed in an analytical balance to compare the mass deviation of the printed dosage forms to achieve the dose required (20 mg NFD).

#### 2.2.4. Imaging

To assess the dimensions, shape, and surface morphology of the 3D printed minitablets, a digital microscope (U500X, CoolingTech, Shenzhen, China) was used, and images were processed by ImageJ v1.46 image analysis software (University of Wisconsin, Madison, WI, USA). Also, dimensions were measured with a calliper (Cole Parmer, Fisher Scientific, Madrid, Spain). To gather a more in-depth analysis of the morphology of the 3D printed minitablets, a scanning electron microscope (JSM 6335F JEOL, Tokyo, Japan) was used at 15.0 kV; the samples were sputter-coated with gold without previous treatment (Q150RS Metalizador QUORUM, Madrid, Spain) for 180 s under vacuum.

#### 2.2.5. Solid-State Characterisation

Solid-state characterisation was performed on 3D printed minitablets, unprocessed excipients, and raw NFD powder. A physical mixture containing the same ratio of active substance and excipients was prepared in an agate mortar and pestle for further analysis. These studies were performed in the CAI technological research centre (Centro de Asistencia a la Investigación, UCM, Madrid, Spain).

##### Fourier-Transform Infrared (FTIR) Spectroscopy

FTIR analysis of printed NFD minitablets was carried out with a Nicolet Nexus 670–870 (Thermofisher, Madrid, Spain). A wavelength range between 400–4000 cm^−1^ was used. Each sample (1–3 mg) was mixed manually in an agate mortar and pestle with KBr (200 mg). The powder mixture was then compressed into compacts using a PerkinElmer hydraulic press set at a pressure of 10 tons for 10 min dwell time. Spectragryph software (version 1.2.9, Oberstdorf, Germany) was used for the interpretation of the spectra.

##### X-ray Powder Diffraction (pXRD)

Powder X-ray analysis was carried out using a Philips^®^ X’Pert-MPD X-ray diffractometer (Malvern Panalytical^®^; Almelo, The Netherlands) equipped with Ni-filtered Cu K radiation (1.54) under 40 kV voltage and a 40 mA current. PXRD patterns were recorded (*n* = 3) at a step scan rate of 0.05° per second from 5° to 40° on the 2-theta scale.

##### Differential Scanning Calorimetry (DSC)

DSC scans were performed using nitrogen as the purge gas on a QA-200 TA instrument (TA instruments, Elstree, UK) calorimeter. NFD minitablets were grinded and weighed (around 10 mg) before being sealed in an open aluminium pan. Temperatures were set to range from 25 to 200 °C at a speed of 10° per minute. The instrument was calibrated using indium as the standard. The glass transition temperatures reported are the transition’s midpoint (*n* = 3).

#### 2.2.6. Dissolution Studies

The dissolution tests were performed in triplicate to evaluate the modified release profile of minitablets using a United States Pharmacopoeia (USP) apparatus 2 (ERWEKA DT 80, Heusenstamm, Germany). A stirring speed of 100 rpm was utilised to investigate the drug release profile under stressed conditions [15]. The employed dissolution media were USP simulated gastric fluid (SGF) without enzymes (pH 1.2) containing 2 g of sodium chloride (NaCl), and 7 mL of hydrochloric acid (HCl, 37%) per litre of deionised water with 0.5% sodium lauryl sulfate for the first two hours, followed by USP simulated intestinal fluid (SIF) without enzymes (pH 6.8) prepared using 6.8 g of monobasic potassium phosphate (KH_2_PO_4_) and 0.62 g of sodium hydroxide (NaOH) per litre of deionised water with 0.5% sodium lauryl sulfate, as described in the USP [16]. SGF (250 mL) was used during the first 2 h. SIF (250 mL) was added and dissolution continued for 22 h at 37 ± 0.5 °C. NaOH (10 M) was used to adjust the pH to 6.8 after the addition of the SIF. Samples (2 mL) were withdrawn from the dissolution media and filtered through a hydrophilic 0.45 μm filter (Millipore, Millex-LCR, Merck, Madrid, Spain) at 5, 10, 15, 30 and 45 min, 1, 1.5, 2, 3, 4, 6, 8, and 24 h. As a comparison, two commercially available tablets of NFD were also tested, Adalat Oros^®^ 30 mg and Adalat Retard^®^ (20 mg).

##### Quantification of NFD by High Performance Liquid Chromatography (HPLC)

Dissolution samples were diluted with HPLC mobile phase (1:1 *v*/*v*) consisting of methanol: water: acetonitrile (36:55:9 *v*/*v*). HPLC analysis was undertaken using a Varian Prostar 230 Solvent Delivery Module, a Varian Prostar autosampler 410, and a Varian Prostar 310 UV-visible detector (Varian, Palo Alto, CA, USA). Integration of the peaks was performed with a Galaxie Chromatography Data System (Varian, CA, USA). NFD was eluted on a Thermo BDS Hypersil C18 reverse-phase column (200 × 4.6 mm, 5 μm). The mobile phase was filtered by a hydrophilic 0.45 μm filter (Millipore, Millex-LCR, Merck, Madrid, Spain), and pumped at a flow rate of 1 mL/min. The sample injection volume was 50 μL. The column temperature was kept at 25 °C, and the detector was set at 240 nm. The method used was previously validated with a detection limit of 0.12 μg/mL, while the quantification limit was 0.4 μg/mL [17].

#### 2.2.7. Data Processing and Statistical Analysis

Statistical analysis for the dissolution study data was performed via a one-way ANOVA test using Minitab v.16 (Minitab Ltd., Coventry, UK) followed by Tukey’s test (95% level of significance). The comparison and modelling of dissolution profiles were performed using the software DDsolver (China Pharmaceutical University, Nanjing, China). The dissolution results were plotted using Origin 2021 (OriginLab Corporation, Northampton, MA, USA).

## 3. Results

### 3.1. Design of Experiments (DoEs)

#### 3.1.1. QbD-Based Model Development and Response Surface Analysis

A full two-level analysis was carried out by a multilinear regression analysis method suggesting that the two-factor interaction models were the best fit for the parameters assessed [18]. The coefficients of the model equations generated for each Critical Quality Attribute (CQA) revealed the goodness of fit of the experimental data to the selected model with high values of R^2^ = 0.9893 and R^2^ = 0.9921 and low *p*-values < 0.01 for both responses, content uniformity, and drug release at 6 h, respectively.

**Figure 1:**
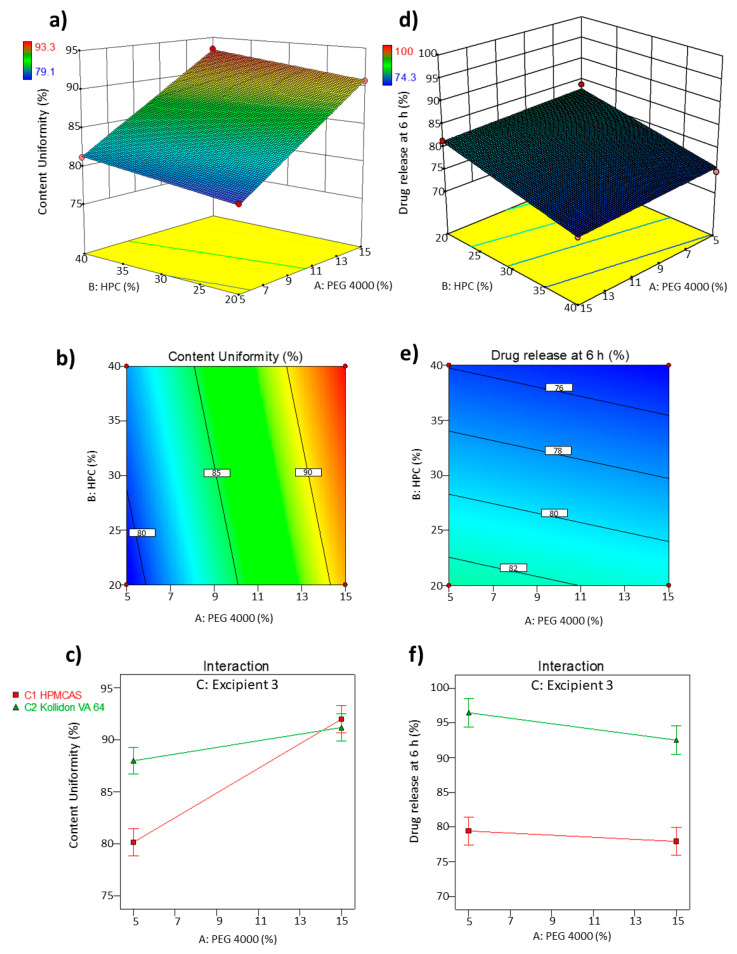
Optimisation of NFD 3D printed minitablets by direct powder extrusion. 3D response surface (**a**,**d**) and 2D contour plots (**b**,**e**) showing the influence of the amount of PEG 4000 and HPC on the content uniformity (%) and drug release at 6 h. Interaction between the excipients (**c**,**f**) HPMCAS and KVA 64 in content uniformity (%) and drug release at 6 h (%).

The 2D-contour plot and 3D-response surface plot, depicted in Figure 1, revealed a higher influence of the concentration of PEG 4000 and the type of modified-release polymer (HPMCAS or KVA64) on content uniformity, whereas the influence of the amount of HPC was found to be negligible. Higher content uniformity was obtained when greater amounts of PEG 4000 and HPMCAS were used (93.2% of NFD loaded on the printed minitablet with 3.4% RSD). The impact on content uniformity for KVA64 was less pronounced (Figure 1c).

Regarding the drug release at 6 h, the most influential variables were the amount of HPC and the type of modified-release polymer (Figure 1e,f). The use of HPMCAS significantly decreased the rate of NFD dissolved at 6 h compared to KVA64. The amount of HPC also played a key role in affecting the drug release, which was reduced when higher amounts of HPC LF grade were used. The amount of PEG 4000 exhibited a negligible effect on the drug release (*p* > 0.05).

#### 3.1.2. Optimal Formulation and Validation of QbD

The search for an optimum formulation was carried out by trading off various CQAs to attain the desired objectives, giving priority to ensuring the uniformity and minimisation of drug release at 6 h. Based on the aforesaid objectives, the optimised process and formulation parameters were: 15% PEG 4000, 40% HPC, and the selection of HPMCAS as the modified-release polymer, resulting in 93.2% content uniformity with a 95% CI (91.1–95.3%) and 74.4% drug release at 6 h with a 95% CI (71.2–77.6%). Validation of the QbD methodology revealed a proximity between the predicted values of the responses with those experimentally obtained (93.7% for content uniformity and 72.5% for drug release at 6 h for prepared check point formulations).

### 3.2. Content Uniformity and Mass Uniformity

The minitablets were designed to contain 20 mg of NFD. The test revealed a high mass uniformity with just a 3.4% RSD in weight (mean = 74.8 ± 2.5 mg). After the optimisation of the formulation was prepared, the content uniformity of NFD inside the 3D printed tablets was also found to be good, with less than 5% drug variability amongst tablets (4.6% RSD, mean = 94.0 ± 4.3%).

### 3.3. Imaging

**Figure 2:**
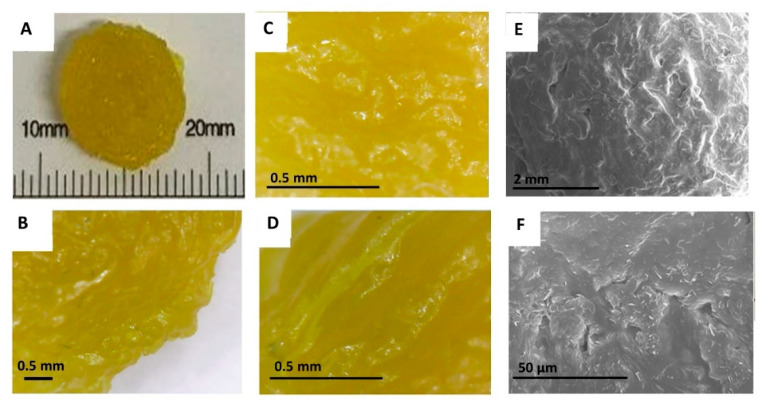
Morphology of NFD 3D printed minitablets. (**A**) Full NFD 3D printed minitablet; (**B**) Image of the 3D printed minitablet side obtained by the digital microscope at 50 magnification. (**C**) Image of the 3D printed minitablet central surface obtained by digital microscope at 100 magnification; (**D**) Image of the 3D printed minitablet side obtained by digital microscope at 100 magnification; (**E**,**F**) Micrographs obtained by Scanning Electronic Microscopy.

The optimised NFD formulation by direct powder extrusion showed a rough transparent yellowish surface as shown in Figure 2. The diameter of the 3D printed minitablet was 7.5 ± 0.1 mm in diameter and 2.5 ± 0.1 mm in height. Several printing defects, such as a porous surface, were observed with the digital microscope both at 50 and 100 magnifications, as well as in the SEM micrographs. The deposition of small crystals on the surface of the tablet was also observed in the SEM micrographs. However, printing defects were not visible to the naked eye and hence, this is unlikely to affect the patient’s adherence to therapy.

### 3.4. Solid-State Characterisation

#### 3.4.1. PXRD

**Figure 3:**
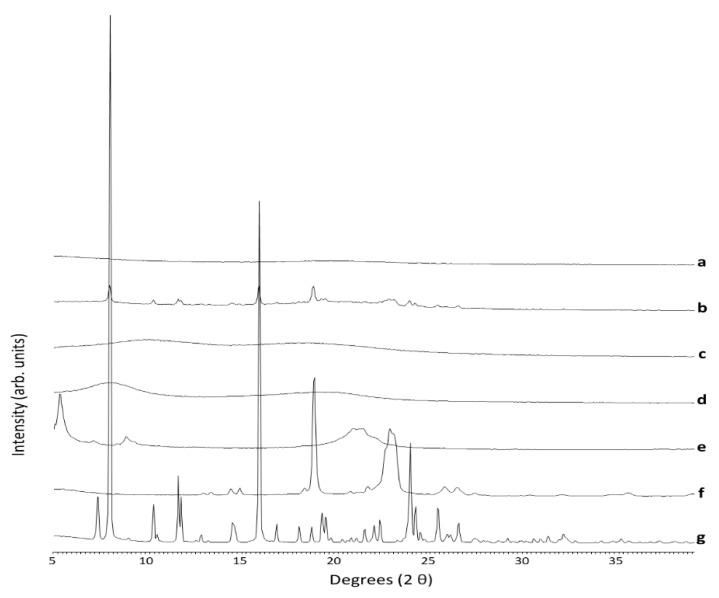
PXRD analysis. Key: (**a**) 3D printed minitablets, (**b**) Physical mixture, (**c**) Unprocessed HPMCAS, (**d**) Unprocessed HPC, (**e**) Unprocessed magnesium stearate, (**f**) Unprocessed PEG 4000, and (**g**) Unprocessed NFD.

The pXRD analysis of the unprocessed materials showed that NFD and PEG 4000 were highly crystalline (Figure 3). The physical mixture of all the components showed Bragg peaks attributed to those of the single components of the mixture, especially NFD and PEG 4000. However, the 3D printed minitablet showed a characteristic amorphous halo (Figure 3a).

#### 3.4.2. Differential Scanning Calorimetry (DSC)

**Figure 4:**
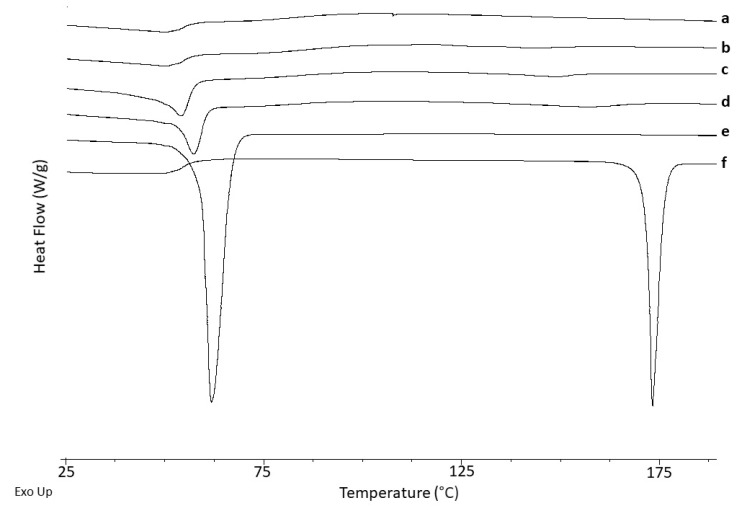
DSC thermograms. Key: (**a**) Unprocessed HPC, (**b**) Unprocessed HPMCAS, (**c**) 3D printed minitablets, (**d**) Physical mixture, (**e**) Unprocessed PEG 4000, and (**f**) Unprocessed NFD.

Unprocessed NFD showed a sharp endothermic event with an onset at 172.2 ± 1.0 °C corresponding to the melting of the drug with a heat of fusion of 110.8 ± 0.8 J/g, (Figure 4 and Table 1). Unprocessed PEG 4000 also exhibited a sharp melting event at 59.2 ± 1.0 °C (Figure 4). The T_g_ for HPMCAS was 123 °C, which is similar to previously reported values [19,20]. HPC did not exhibit any T_g_ in the thermograms at the conditions tested, bearing in mind its low T_g_ temperature, below 0 °C [21].

Based on the XRD results, in which a characteristic amorphous halo was observed for the 3D printed minitablet, it is apparent that NFD recrystallises during the DSC heating ramp, which has been previously reported [13]. A single T_g_ was observed for the 3D printed minitablet at 49.7 ± 0.3 °C. However, the T_g_ of unprocessed NFD after melt quenching has been reported to be 46.2 °C [13]. This indicates that a homogeneous amorphous domain has probably been formed between the HMPCAS (T_g_ = 123 °C) and HPC (T_g_ < 0 °C) [19,20,21,22] with the active ingredient. Even though physical stability studies should be performed, the low T_g_ of the composite indicates that it is likely not to be a highly stable amorphous formulation, which can explain the crystallisation upon exposure to heating in the DSC, with a depressed melting point at 137.4 ± 0.6 °C and enthalpy of fusion of 3.5 J/g [13].

#### 3.4.3. Fourier Transform Infrared Spectroscopy (FTIR)

**Figure 5:**
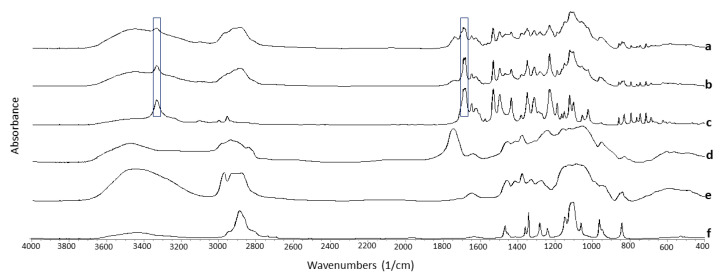
FTIR spectra. Key: (**a**) 3D printed minitablets, (**b**) Physical mixture, (**c**) Unprocessed NFD, (**d**) Unprocessed HPMCAS, (**e**) Unprocessed HPC, and (**f**) Unprocessed PEG 4000. Peak shifts are highlighted within boxes.

FTIR spectra showed hydrogen-bonding interactions between NFD and the excipients (Table 2 and Figure 5). Shifts were observed in the C-H (3333 cm^−1^) and the carbonyl group C=O (1690 cm^−1^) functional groups of NFD after 3D printing.

These shifts were not observed in the physical mixture, indicating that the hot-melt extrusion process triggers the interaction by H-bond formation between the NFD and the excipients, leading to the formation of a single T_g_ amorphous composite. Also, the 3D printed minitablet showed broader bands, probably due to its amorphous nature, compared to the physical mixture and the unprocessed NFD.

### 3.5. Dissolution Studies

**Figure 6:**
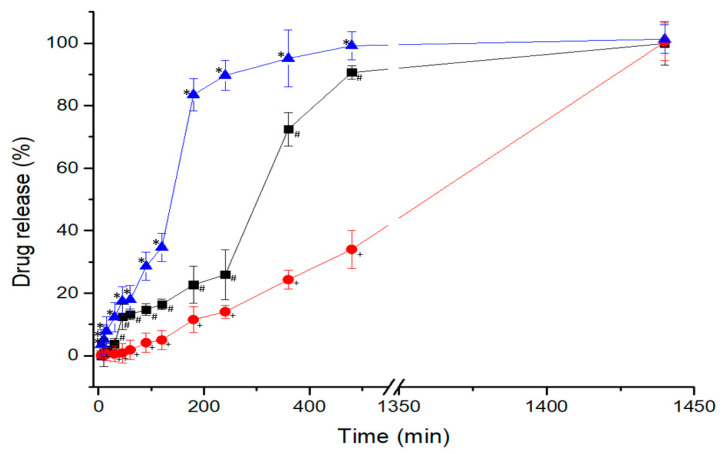
Dissolution profile for the 3D printed minitablets formulation (-■-) compared to the commercially marketed NFD tablets: Adalat Oros^®^ 30 mg (-●-) and Adalat Retard^®^ 20 mg (-▲-). Key: + *p* < 0.05 Adalat Oros^®^ vs. all the other formulations (including t = 15 min); # *p* < 0.05 3D printed minitablets formulation vs. the marketed formulations (including t = 15 min) and * *p* < 0.05 Adalat Retard^®^ vs. all the other formulations.

As NFD is a crystalline-nature very poorly water-soluble compound, dissolution studies were performed using sodium lauryl sulphate to ensure sink conditions [23,24]. Two marketed NFD tablets were used to compare the dissolution profile of the 3D printed formulation, resulting in marked differences between them (Figure 6). Adalat Oros^®^ is an osmotic system consisting of a semi-impermeable barrier created mainly by cellulose acetate and a dual reservoir with sodium chloride and NFD amongst other excipients [25]. The compartment with sodium chloride, upon wetting, increases the osmotic pressure of the system, forcing the NFD contained in the other compartment to be slowly released across the pore located at the surface of the tablet. This behaviour resulted in a zero-order release (R^2^ = 0.9958), as observed in Figure 6.

However, the NFD from the Adalat Retard^®^ tablets showed a dissolution profile characterised by a faster disintegration of the tablet into smaller fragments, resulting in a marked burst effect with drug release greater than 80% at 3 h. The 3D printed minitablet containing HPMCAS showed a significantly different (*p* < 0.05) intermediate release profile between Adalat Oros^®^ and the Adalat retard^®^ formulation, exhibiting a sustained release over 4 h with less than 30% drug release, followed by a faster release up to 24 h, reaching 99.9% drug released. The 3D printed minitablet did not show any disintegration into smaller fragments, but just erosion from the surface becoming smaller in size over time. These results indicate that the kinetics that govern the dissolution of Adalat Retard^®^ and 3D printed minitablets follow first order (R^2^ = 0.9387) and Hixson-Crowell (R^2^ = 0.9293), respectively.

## 4. Discussion

In this work, we have demonstrated that direct powder extrusion can be successfully utilised to manufacture high drug-loaded solid dosage forms (25% of active ingredient) in a single step, which is useful for implementation in clinical settings by healthcare professionals such as clinical pharmacists. The content uniformity of the optimised dosage form was above 90%, with low variability (< 5%). The time for printing each tablet was low (2 min), which allows the preparation of a personalised antihypertensive treatment adapted to the patient’s need (30 tablets, i.e., monthly supply) in one hour.

Direct powder extrusion can trigger intimate contact between drug and excipients, leading to hydrogen bond formation and amorphous solid dispersions overcoming solubility issues of poorly water-soluble drugs. Long-term stability studies are not required, considering that medication is prepared for immediate use (<7 days), which reduces the issue of physical instability of amorphous systems, especially if tablets are packed within aluminium blisters. In addition, the small size and weight (~80 mg) of the 3D printed minitablets allow them to be easily combined inside a 0-size capsule with other minitablets containing other active ingredients, which would allow preparation of a multidrug solid dosage form with minimal incidence of physicochemical interactions between drugs, as each one is contained in an independent tablet. Incorporating the minitablets into capsules minimises the potential difficulty or discomfort in swallowing occurring due to the lack of smoothness of the minitablet surface, even if this is not visible to the naked eye, to ensure that it does negatively impact on patient acceptability and adherence. This roughness can be attributed to the molecular weight (95 KDa) and particle size of the HPC utilised, and as described by other authors, the smoothness of the tablets can increase by reducing the molecular weight of the HPC utilised for printing [12,26]. When high molecular HPC as L grade (140 KDa) is used instead, some authors have described lower drug release compared to other HPC with low molecular weight, and this is why this was not selected in our studies [12].

Dissolution is key, and can be controlled by the combination of excipients employed. Quality by design experiments informed the optimal excipient percentages needed to fabricate sustained-release tablets. This is especially important for antihypertensive drugs such as NFD, in which a marked burst effect can worsen orthostatic hypotension in the morning, and fine control of blood pressure is desired over prolonged periods. The 3D printed minitablets obtained by direct powder extrusion have shown a better release profile to treat hypertension, with controlled release over 24 h, compared to other commercially available formulations such as Adalat Retard^®^ tablets. The core of the latter consists of microcrystalline cellulose, corn starch, lactose, polysorbate 80, magnesium stearate, hypromellose, and macrogol 4000 [14]. Even though modified-release excipients are used, the tablet disintegrates faster into smaller fragments upon getting in contact with gastric fluids (first-order release). However, the 3D printed minitablet, despite its small size, maintains its geometry intact during the whole dissolution experiment, and erosion is only visible over time. This release behaviour is explained by the Hixson-Crowell kinetic model, which considers that dissolution occurs in planes parallel to the surface of the drug when the tablet dimensions decrease proportionally without altering the tablet geometry [27]. The combination of PEG 4000 (15%), HPC (40%), and HPMCAS (19%) has led to the sustained release of NFD over 24 h. HPMCAS performed better than KVA64 in terms of controlled drug release. Greater amounts of a hydrophilic matrix rate-controlling polymer (HPC) resulted in slower drug release. The selected HPC was LF grade with a molecular weight of 95 KDa [28,29]. Lower-molecular-weight HPC up to 370 KDa undergoes significant erosion, while diffusion through the swelling gel layer becomes more significant in polymers with a molecular weight above this threshold [30]. These characteristics have been shown previously in tablets fabricated by conventional methods, as HPC possesses good compactability properties. However, in this work, this effect has been demonstrated in tablets manufactured by 3D printed direct powder extrusion [31]. In addition, the thermoplastic behaviour of HPC conferred optimal properties to be moulded and extruded in situ with the NFD and the other two excipients, resulting in adequate melt flow, which resulted in the formation of a successful amorphous solid dispersion. The combination of HPC and HPMCAS was key in allowing complete release in a 24 h period. Slow and incomplete dissolution profiles have been previously reported for 3D printed solid dosage forms, especially when high levels of hydroxypropyl methylcellulose were used [7], which is not suitable, as the oral bioavailability of the active ingredient would be reduced.

## 5. Conclusions

Direct powder extrusion has been shown to be a suitable technique for single-step manufacturing of small tablets (<100 mg) with high drug loading (25%). The designs of experiments are key for finding the optimal excipient combination, especially when a sustained drug release is desirable. 3D printed NFD minitablets containing 20 mg were manufactured combining 15% PEG 4000, 40% HPC, 19% HMPCAS, and 1% magnesium stearate. The printed minitablets exhibited a clinically preferred release profile compared to commercially available formulations, such as Adalat Retard^®^, which disintegrated too fast, resulting in a marked burst effect. However, the structure of the 3D printed minitablet, considering its small size, remained intact over prolonged periods, allowing for a controlled drug release for 24 h based on erosion.

## Figures and Tables

**Table 1:** Enthalpy of fusion, melting events, and glass transition for NFD 3D printed mini-tablets, and unprocessed materials and physical mixture.

Sample	Enthalpy of Fusion (J/g)	Melting (Onset) (°C)	Glass Transition (°C)
3D printed mini-tablets	7.9 ± 0.2 3.5 ± 0.4	51.3 ± 0.9 137.4 ± 0.6	49.7 ± 0.3
Raw NFD	110.8 ± 0.8	172.2 ± 1.0	-
Physical Mixture	28.41 ± 0.6 6.2 ± 0.8	53.2 ± 0.5 142.4 ± 0.9	-
PEG 4000	191.9 ± 0.7	59.2 ± 1.0	-
HPMCAS	-	-	122.6 ± 0.7
HPC	-	-	-

**Table 2:** FTIR spectra of the 3D printed minitablets, the raw NFD, and the physical mixture. Bands that showed a characteristic shift are illustrated in the Table.

Sample	C=O (cm^−1^)	C-H (cm^−1^)
3D printed mini-tablets	1690	3333
Raw NFD	1682	3332
Physical Mixture	1680	3332
PEG 4000	N/A	N/A
HPMCAS	N/A	N/A
HPC	N/A	N/A

## Data Availability

Data is contained within the article.

## References

[1] Okafor-Muo O.L., Hassanin H., Kayyali R., ElShaer A. (2020). 3D Printing of Solid Oral Dosage Forms: Numerous Challenges with Unique Opportunities. J. Pharm. Sci..

[2] Harvey A., Brand A., Holgate S.T., Kristiansen L.V., Lehrach H., Palotie A., Prainsack B. (2012). The future of technologies for personalised medicine. New Biotechnol..

[3] Tiboni M., Campana R., Frangipani E., Casettari L. (2021). 3D printed clotrimazole intravaginal ring for the treatment of recurrent vaginal candidiasis. Int. J. Pharm..

[4] Serrano D.R., Terres M.C., Lalatsa A. (2018). Applications of 3D printing in cancer. J. 3D Print. Med..

[5] Konta A.A., Garcia-Pina M., Serrano D.R. (2017). Personalised 3D Printed Medicines: Which Techniques and Polymers Are More Successful?. Bioengineering.

[6] Tyson R.J., Park C.C., Powell J.R., Patterson J.H., Weiner D., Watkins P.B., Gonzalez D. (2020). Precision Dosing Priority Criteria: Drug, Disease, and Patient Population Variables. Front. Pharmacol..

[7] Khaled S.A., Burley J., Alexander M., Yang J., Roberts C.J. (2015). 3D printing of tablets containing multiple drugs with defined release profiles. Int. J. Pharm..

[8] WHO Cardiovascular Diseases 05/2017. https://www.who.int/health-topics/cardiovascular-diseases/#tab=tab_1.

[9] Brown M.T., Bussell J.K. (2011). Medication adherence: WHO cares?. Mayo Clin. Proc..

[10] Klobusicky J.J., Aryasomayajula A., Marko N. (2015). Evolving Patient Compliance Trends: Integrating Clinical, Insurance, and Extrapolated Socioeconomic Data. AMIA Annu. Symp. Proc..

[11] Fina F., Madla C.M., Goyanes A., Zhang J., Gaisford S., Basit A.W. (2018). Fabricating 3D printed orally disintegrating printlets using selective laser sintering. Int. J. Pharm..

[12] Goyanes A., Allahham N., Trenfield S.J., Stoyanov E., Gaisford S., Basit A.W. (2019). Direct powder extrusion 3D printing: Fabrication of drug products using a novel single-step process. Int. J. Pharm..

[13] Ayyoubi S., Cerda J.R., Fernández-García R., Knief P., Lalatsa A., Healy A.M., Serrano D.R. (2021). 3D printed spherical mini-tablets: Geometry versus composition effects in controlling dissolution from personalised solid dosage forms. Int. J. Pharm..

[14] Agencia Española de Medicamentos y Productos Sanitarios Ficha Técnica Adalat Oros® 30 mg. https://cima.aemps.es/cima/publico/detalle.html?nregistro=59538.

[15] U.S.P. (2021). <711> Dissolution.

[16] U.S.P. (2021). Reagents: Test Solutions.

[17] Cerda J.R., Arifi T., Ayyoubi S., Knief P., Ballesteros M.P., Keeble W., Barbu E., Healy A.M., Lalatsa A., Serrano D.R. (2020). Personalised 3D Printed Medicines: Optimising Material Properties for Successful Passive Diffusion Loading of Filaments for Fused Deposition Modelling of Solid Dosage Forms. Pharmaceutics.

[18] Serrano D.R., Walsh D., O’Connell P., Mugheirbi N.A., Worku Z.A., Bolas-Fernandez F., Galiana C., Dea-Ayuela M.A., Healy A.M. (2018). Optimising the in vitro and in vivo performance of oral cocrystal formulations via spray coating. Eur. J. Pharm. Biopharm..

[19] Sturm D.R., Danner R.P., Moser J.D., Chiu S.W. (2019). Application of the Vrentas–Duda free-volume theory of diffusion below the glass-transition temperature: Application to hypromellose acetate succinate–solvent systems. J. Appl. Polym. Sci..

[20] Sarabu S., Kallakunta V.R., Bandari S., Batra A., Bi V., Durig T., Zhang F., Repka M.A. (2020). Hypromellose acetate succinate based amorphous solid dispersions via hot melt extrusion: Effect of drug physicochemical properties. Carbohydr. Polym..

[21] Picker-Freyer K.M., Durig T. (2007). Physical mechanical and tablet formation properties of hydroxypropylcellulose: In pure form and in mixtures. AAPS PharmSciTech.

[22] Kallakunta V.R., Sarabu S., Bandari S., Batra A., Bi V., Durig T., Repka M.A. (2020). Stable amorphous solid dispersions of fenofibrate using hot melt extrusion technology: Effect of formulation and process parameters for a low glass transition temperature drug. J. Drug Deliv. Sci. Technol..

[23] Emara L.H., Badr R.M., Elbary A.A. (2002). Improving the dissolution and bioavailability of nifedipine using solid dispersions and solubilizers. Drug Dev. Ind. Pharm..

[24] British Pharmacopeia (2020). Dissolution. https://www.pharmacopoeia.com/the-british-pharmacopoeia.

[25] Liu X., Chen D., Zhang R. (2003). Evaluation of monolithic osmotic tablet system for nifedipine delivery in vitro and in vivo. Drug Dev. Ind. Pharm..

[26] Serrano D.R., Fernandez-Garcia R., Mele M., Healy A.M., Lalatsa A. (2019). Designing Fast-Dissolving Orodispersible Films of Amphotericin B for Oropharyngeal Candidiasis. Pharmaceutics.

[27] Bruschi M.L. (2015). Mathematical models of drug release. Strategies to Modify the Drug Release from Pharmaceutical Systems.

[28] Teixeira A.Z.A. (2009). Hydroxypropylcellulose Controlled Release Tablet Matrix Prepared by Wet Granulation: Effect of Powder Properties and Polymer Composition. Braz. Arch. Biol. Technol..

[29] Dürig T., Lusvardi K.M., Harcum W.W. (2011). Hydroxypropylcellulose in Modified Release Matrix Systems: Polymer Molecular Weight Controls Drug Release Rated and Mechanism Ashland.

[30] (2017). Ashland-KlucelTM Hydroxypropylcellulose—Physical and Chemical Properties. https://www.ashland.com/file_source/Ashland/Product/Documents/Pharmaceutical/PC_11229_Klucel_HPC.pdf.

[31] Roy D.S., Rohera B.D. (2002). Comparative evaluation of rate of hydration and matrix erosion of HEC and HPC and study of drug release from their matrices. Eur. J. Pharm. Sci..

